# Effect of an acute exercise on early responses of iron and iron regulatory proteins in young female basketball players

**DOI:** 10.1186/s13102-022-00465-7

**Published:** 2022-04-15

**Authors:** Justyna Cichoń, Joanna Ostapiuk-Karolczuk, Mirosława Cieślicka, Hanna Dziewiecka, Anita Marcinkiewicz, Małgorzata Tafil-Klawe, Piotr Basta, Dariusz Maciejewski, Anna Skarpańska-Stejnborn

**Affiliations:** 1 Department of Biological Sciences, Faculty of Physical Culture in Gorzow Wielkopolski, Poznan University of Physical Education, Estkowskiego 13, 66-400 Gorzów, Wielkopolski Poland; 2 Department of Physiology, Collegium Medicum in Bydgoszcz, Nicolaus Copernicus University in Toruń, M. Skłodowskiej-Curie 9, 85-094 Bydgoszcz, Poland; 3 Department of Physical Education and Sport, Poznań University of Physical Education, Faculty of Physical Culture in Gorzów Wielkopolski, Estkowskiego 13, 66-400 Gorzów, Wielkopolski Poland

**Keywords:** Iron homeostasis, Hepcidin, IL-6, Ferritin, Transferrin, Acute exercise, Female athletes

## Abstract

**Background:**

The accumulation of physiological stress and the presence of inflammation disturb iron management in athletes during intense training. However, little is known about the mechanisms regulating iron levels in athletes during training periods with low training loads. In the current study, we analyzed the effect of an acute exercise on early responses of iron and iron regulatory proteins at the end of such training periods.

**Methods:**

The study was performed at the end of competitive phase of training. A total of 27 trained female basketball players were included in the study after application of the inclusion/exclusion criteria. The participants performed an incremental exercise on a treadmill. Blood samples were taken before the test, immediately after exercise, and after 3 h of restitution. Parameters, such as interleukin (IL) 6, hepcidin, ferritin, transferrin, hemopexin, and lactoferrin levels, total iron-biding capacity (TIBC), unsaturated iron-biding capacity (UIBC) were determined by using appropriate biochemical tests.

**Results:**

The level of iron increased significantly after exercise, and then decreased within next 3 h restitution. Except for iron levels, only TIBC levels significantly increased after exercise and decreased to baseline level during rest period. No significant changes in the levels of hepcidin, IL-6, and other proteins related to the iron homeostasis were observed.

**Conclusions:**

The increases in iron level after acute exercise is short-term and transient and appear to have been insufficient to induce the acute systemic effects in rested athletes.

## Background

Iron is fundamentally important for the optimal functioning of athletes with implications for sports performance. As it is a component of hemoglobin, myoglobin, cytochrome, and various muscle cells enzymes it plays a vital role in transporting oxygen, energy metabolism, supporting the immune system [1].

Athletes experience disturbances of systemic iron homeostasis, especially during training periods characterized by intense training loads. ​Based on the available data, the risk of depletion of iron stores is especially high in women and adolescents, which may lead to severe iron deficiency. Insufficient reserves of iron in the body can reduce athletic performance, which may be manifested as fatigue, exercise intolerance, or even cognitive function impairment [2–4]

The presence of iron deficiency in physically active women, especially endurance athletes, has been a subject of attention in recent years. This is due to the particularly high frequency of iron deficiency in female players. In some cases, their iron levels are even twice as low as in non-trained humans. This problem is best illustrated by the research of Pate et al. [5], in which 213 female players with various types of physical activity were observed. The results showed that in physically active women the concentration of iron was lower, but also the negative correlation between ferritin level and level of physical activity was observed in both recreational active women and elite athletes [6]. In a large cross-sectional study on both female and male athletes, the relationship between the parameters of iron management depending on the type of physical effort (aerobic, anaerobic) was assessed. The authors reported that female players practicing sports requiring mixed energy sources (i.e., aerobic and anaerobic), such as volleyball, handball, or athletics, had the highest risk of iron deficiency and disturbances of its economy, compared to the same efforts considered separately [7].

Moreover, Auersperger et al. [4] have shown cumulative effects of regularly performed exercise training on the iron and iron regulatory proteins pointing out that iron stores, as well as hepcidin levels, may decrease during a long-term training and did not recover after 10 days. Research also indicates that the negative effects of intense physical activity are deeper in less trained players than in highly trained athletes [8].

In contrast, only a few studies have looked at the acute post-exercise response of iron and iron-responsive proteins. Most of them indicate an elevated level of hepcidin 24 h after the exercise, which was preceded by a sharp increase in serum iron, accompanied by an increase in inflammatory markers [9, 10].

Further, based on the available data, the iron indices and hematological parameters in elite athletes change during the training cycle, depending on the training intensity and frequency [11–13]. Malcovati et al. [14] assessed the effect of exercise and training phase on hematological parameters and iron homeostasis indicators in 923 professional football players. They found that in well-trained athletes, most variables [e.g., ferritin, hemoglobin, and mean corpuscular volume (MCV)] were highest at the start of the playing season and then decreased. It seems that the direction of changes in hematological parameters and indicators of serum iron homeostasis after exercise is determined by the training loads used in individual training cycles. However, as mentioned above, most of the studies conducted to date have focused on the competition period, where an increase in inflammation is observed, which may greatly affect post-exercise iron management disorders [15, 16]. Little is known about whether these changes are also sustained at the end of the competition period, when the training loads are greatly reduced.

Several mechanisms may be responsible for increasing serum iron concentration in response to acute exercise including hemolysis, hematuria, and gastrointestinal bleeding [17]. Oxidative stress induced by exercise and the associated inflammation result in biochemical changes in the erythrocyte cell membrane, leading to its increased susceptibility to damage and increased intravascular hemolysis [18]. According to Barros et al. [19], hemolysis is not the only source of the increased concentration of iron ions in serum during intense exercise. They showed that in individuals with elevated after exercise iron concentrations, only approximately 20% of iron ions in serum come from hemolysis. The authors suggested that ferritin and transferrin are the main source of iron ions in this case. Also, literature data demonstrated that free oxygen radicals (reactive oxygen species, ROS) are primarily responsible for the release of iron ions from ferritin [20].

The post-exercise iron homeostasis is regulated by several factors including transferrin and ferritin which may bind nearly all iron circulating in the serum. Under physiological conditions, this chelation makes the iron soluble, prevents iron-mediated free-radical toxicity, and facilitates the transport of iron into cells. Hence, the total iron-binding capacity (TIBC) of serum reflects serum transferrin levels [21, 22].

A major role in the regulation of post-exercise iron management plays also interleukin (IL) 6. IL-6 is a key regulator of not only the inflammatory response but also the activity of the iron-regulating hormone hepcidin [23]. This hormone adversely affects the activity of the main iron export protein, ferroportin, which leads to a decrease of iron absorption in the intestine, and inhibition of iron release from hemolyzed red blood cells and internal organs that store iron [24].

Also, hemopexin and lactoferrin are involved in the regulation of iron levels. They capture free heme or iron ions, and thus contribute to the regulation of inflammation [25, 26]. Both proteins reduce macrophage production of pro-inflammatory cytokines, including IL-6. They thus affect the activity of ferroportin – positively; and ferritin – negatively, preventing intracellular iron overload and inhibiting the development of inflammation [26].

To date, studies into the changes in iron levels or the levels of proteins responsible for the regulation of iron homeostasis have primarily focused on pre- and post-exercise values and, predominantly, on the restitution period 24 h post-exercise [4, 15, 16]. Only few studies have focused on the early restitution period. Such studies mainly consider the relationship between iron status and the levels of hepcidin [9, 39]. However, as a caveat to interpreting the results of such studies, the reported data hold for athletes during periods of high-stress training, and the observed levels of iron metabolism markers were not compared with those in completely rested athletes.

Further, based on the available data, the iron indices and hematological parameters in elite athletes change during the training cycle, depending on the training intensity and frequency [11–13]. Malcovati et al. [14] assessed the effect of exercise and training phase on hematological parameters and iron homeostasis indicators in 923 professional football players. They found that in well-trained athletes, most variables [e.g., ferritin, hemoglobin, and mean corpuscular volume (MCV)] were highest at the start of the playing season and then decreased. It seems that the direction of changes in hematological parameters and indicators of serum iron homeostasis after exercise is determined by the training loads used in individual training cycles. However, as mentioned above, most of the studies conducted to date have focused on the competition period, where an increase in inflammation is observed, which may greatly affect post-exercise iron management disorders [15, 16]. Little is known about whether these changes are also sustained at the end of the competition period, when the training loads are greatly reduced, and rest and recovery are emphasized.

We hypothesized that the changes in the level of iron and its regulatory proteins may depend not only on the acuity of exercise but also on the training load. Accordingly, the current study aimed to assess the impact of acute exercise performed at the end of the competition period on iron levels and selected parameters related to the regulation of serum iron homeostasis in trained basketball players.

## Methods

### Participants and study design

Twenty-seven female trained basketball players participated in the study. Inclusion criteria consisted of female athletes participant (minimum of 6 years of training experience), free from iron homeostasis disturbances (e.g., iron deficiency, anemia), with regular menstruation cycle. Exclusion criteria consisted of: the presence of acute or chronic inflammation of pain disability, fever, infections, iron supplementation, use of any anti-inflammatory drugs. All participants belong to the youth groups of ENEA AZS AJP Gorzów Wielkopolski (1st League and Premier League). Table 1 shows the anthropometric characteristics of the study participants. The research was conducted at the end of the competition period, two weeks after the last competition games. All players were on the same team and were subjected to the same training regime.

**Table 1:** Basic characteristics of study participants (n = 27)

Parameter	x̅ ± SD
Age (years)	16.55 ± 0.96
Body mass (kg)	66.40 ± 13.68
Height (cm)	173.45 ± 5.14
Training internship (years)	7.3 ± 1.2

Values are presented as the mean ± SD

### Experimental design

**Fig. 1:**
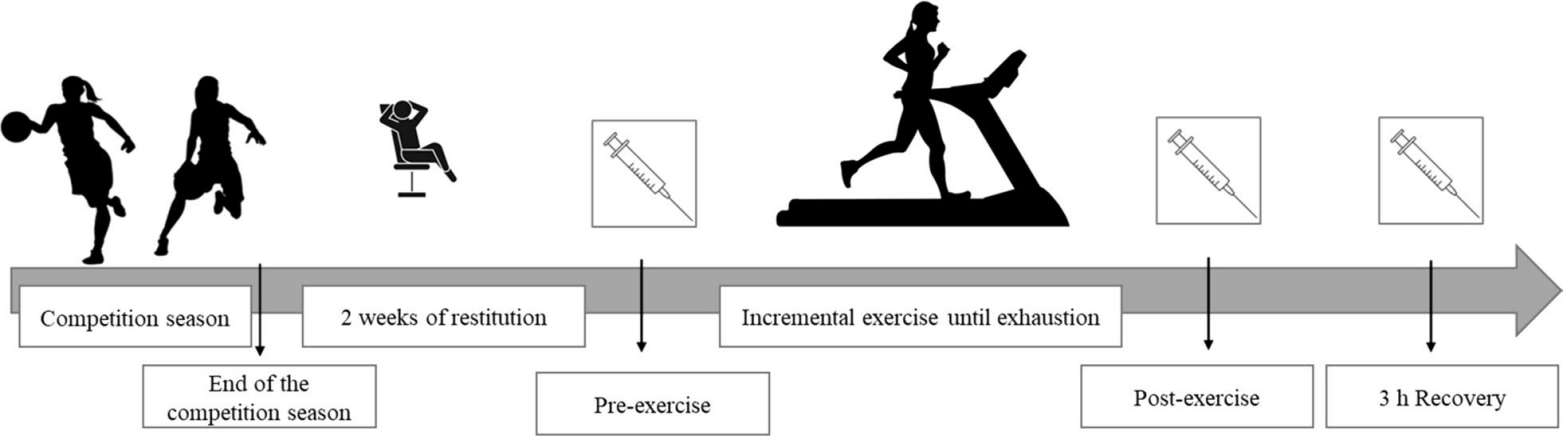
Schematic illustration of the study design and experimental timeline

In this study, the participants performed acute exercise until exhaustion. The experiment was designed for assessing the disturbances in iron homeostasis caused by an acute exercise thus biochemical assays were performed using blood collected at three time-points: at rest (Pre-exercise), immediately after the exercise (post-exercise) and after 3 h of rest (3 h Recovery) (Fig. 1). All participants performed an incremental exercise test on HP Cosmos Treadmill (serial no. cos30004va04; Nussdorf—Traunstein Germany). The test protocol was as follows: the starting speed of the treadmill for the participants was 8.0 km/h; it was then increased every 2 min by 1.0 km/h, until exhaustion. The test ended with voluntary exhaustion of the subjects.

During the test, minute ventilation (VE), oxygen uptake (VO_2_), and carbon dioxide production (VCO_2_) were determined continuously using the exhaled air analyzer MES (measurement system contains of a pneumotach headpiece patented by MES and fast analyzers of carbon dioxide and oxygen fit for applying "breath-by-breath" method) to each exhalatory phase. Table 2 shows the basic characteristics of the exercise. The participants were verbally encouraged to continue for as long as possible. Heart rate (bpm) was recorded using a sport tester (Polar PE 3000). This model of acute exercise until exhaustion is often used in research focused on the observation of changes in biochemical parameters in response to the short-term but very intense effort [16].

**Table 2:** Basic characteristics of exercise

Parameter	x̅ ± SD
HR_max_ (Bpm)	192.13 ± 12.29
Test duration (s)	634.33 ± 96.18
V_max_ ( km x h^−1^)	12.85 ± 1.14
VO_2max_ (ml x kg^−1^ × min^−1^)	51.46 ± 8.41
RER	0.97 ± 0.11

Values are presented as the mean ± SD; HR_max_, maximal heart rate; V_max_, maximal run velocity; VO_2max_, maximal oxygen consumption; RER (VCO_2_/VO_2_), respiratory exchange ratio

### Blood analysis

Hematological variables, such as red blood cell count (RBC), hemoglobin (Hb), hematocrit (HCT), mean corpuscular hemoglobin (MCH), mean corpuscular hemoglobin concentration (MCHC), mean corpuscular volume (MCV), red cell distribution width (RDW), mean platelet volume (MPV), and platelet count (PLT), were analyzed using the MYTHIC 18 Haematology Analyser (Orphee Medical, Geneva, Switzerland). Relative changes in plasma volume were calculated from blood hematocrit and hemoglobin concentrations using Dill and Costill’s equation (Dill, Costil, 1974). Iron concentration and TIBC (Total Iron-Binding Capacity) were determined using colorimetric method with chromogens (cat. no. 1–418–01–50 and 1–421–0060, respectively; BioMaxima, Lublin, Poland). Unsaturated iron-binding capacity (UIBC) was calculated using the formula: UIBC = TIBC – Fe. Transferrin saturation was calculated as serum iron/TIBC. Enzyme-linked immunosorbent assay kits and Thermo Scientific Multiscan GO microplate spectrophotometer (Fisher Scientific, Vantaa, Finland) were used to determine serum levels of the following molecules: IL-6, transferrin, hemopexin, and lactate dehydrogenase (LDH) (SunRed Biotechnology Co., Ltd (Shanghai, China); hepcidin, myoglobin, and ferritin (DRG International Inc., Springfield, New York, USA); and lactoferrin (AssyPro, St Charles, MO, USA).

### Statistical analysis

Statistical analyses were performed using STATISTICA v. 13.0 software package (Stat–Soft, Kraków, Poland). Descriptive statistics, including mean and SD, were used to visualize immediate trends and patterns. Shapiro–Wilk test was used to examine the normal distribution of variables. Levene’s test was used to verify the homogeneity of variance. Lactoferrin level was not normally distributed, and was log transformed for further analysis. One-way analysis of variance with repeated measures (ANOVA), with Tukey's *post-hoc* analysis was used to asses differences in measured variables of the three assessments points (pre-exercise, post-exercise and 3 h recovery) respectively. As a measure of effect size Cohen`s d was calculated. Using Cohen`s criteria, effect size was interpreted as small (0.2), moderate (0.5), large (0,8) (Cohen, 1988). For correlation analysis, Pearson’s coefficient of linear correlation was calculated. The level of significance for all analysis was set at *p* ≤ 0.05.

## Results

**Fig. 2:**
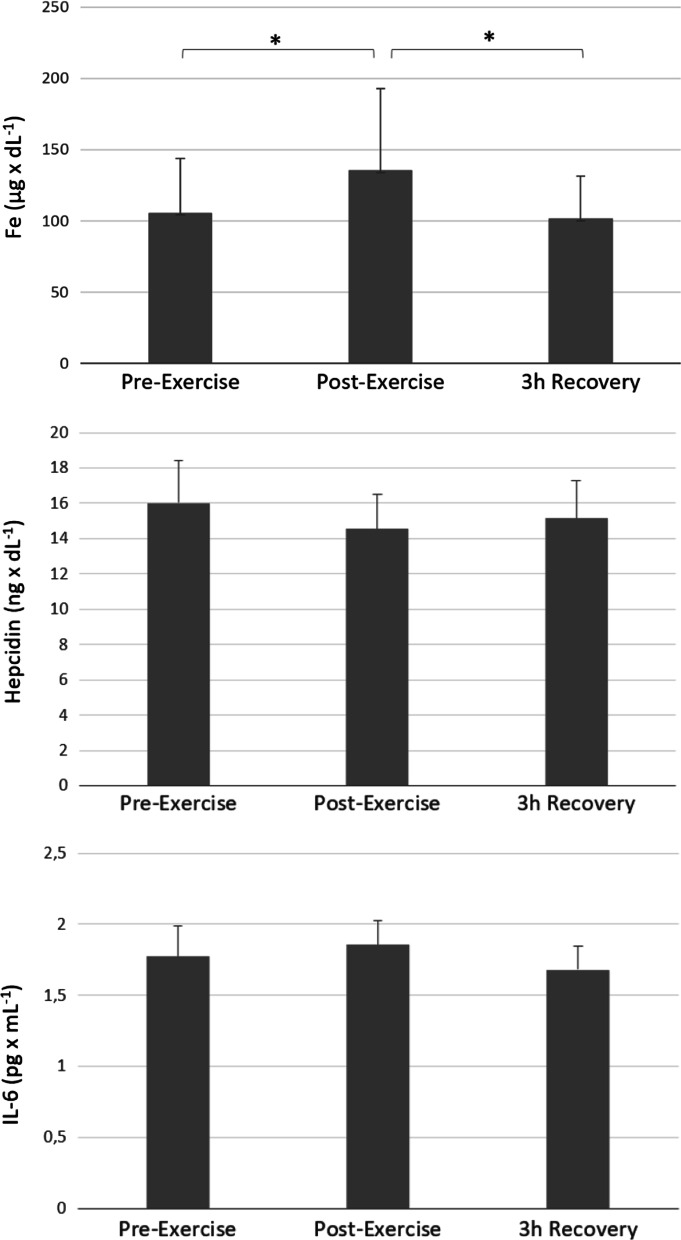
The effect of intense exercise on iron, hepcidin and IL-6 plasma concentration. *Note.* Values are presented as mean ± SD; Significant differences *p < .05

Iron serum concentration significantly increased after exercise (*p* < 0.05, Cohen`s d = 0.62, Pre-exercise vs. Post-exercise); in the following 3 h of restitution, the iron level decreased and reached values close to the baseline. No significant changes in the serum concentration of hepcidin and IL-6 were observed during the study (Fig. 2, Table 2).

**Fig. 3:**
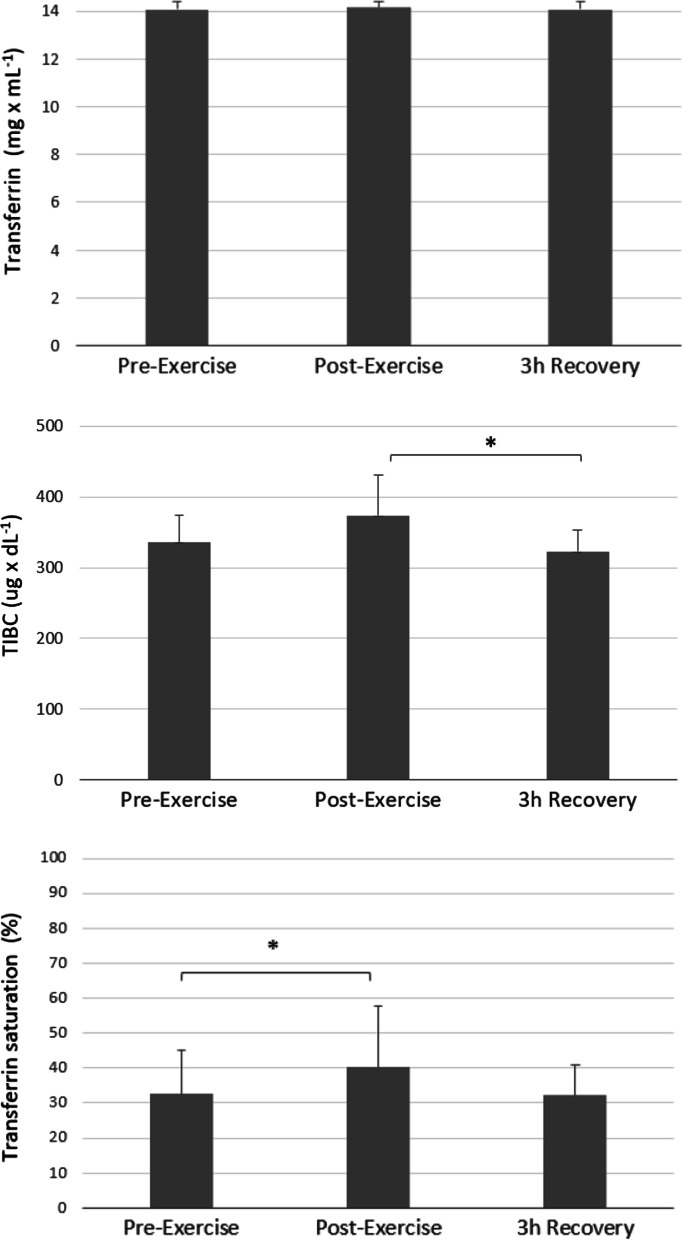
The effect of intense exercise on transferrin, TIBC and transferrin saturation. *Note.* Values are presented as mean ± SD; Significant differences *p < .05

No significant changes in transferrin levels were observed; however, TIBC slightly increased after exercise and then significantly decreased in the 3 h after exercise (*p* < 0.05, Cohen`s d = 0.64, post-exercise vs. 3 h Recovery). Transferrin saturation significantly increased immediately after exercise (*p* < 0.05, Cohen`s d = 0.17, Pre-exercise vs. Post-exercise) (Fig. 3, Table 2).

**Fig. 4:**
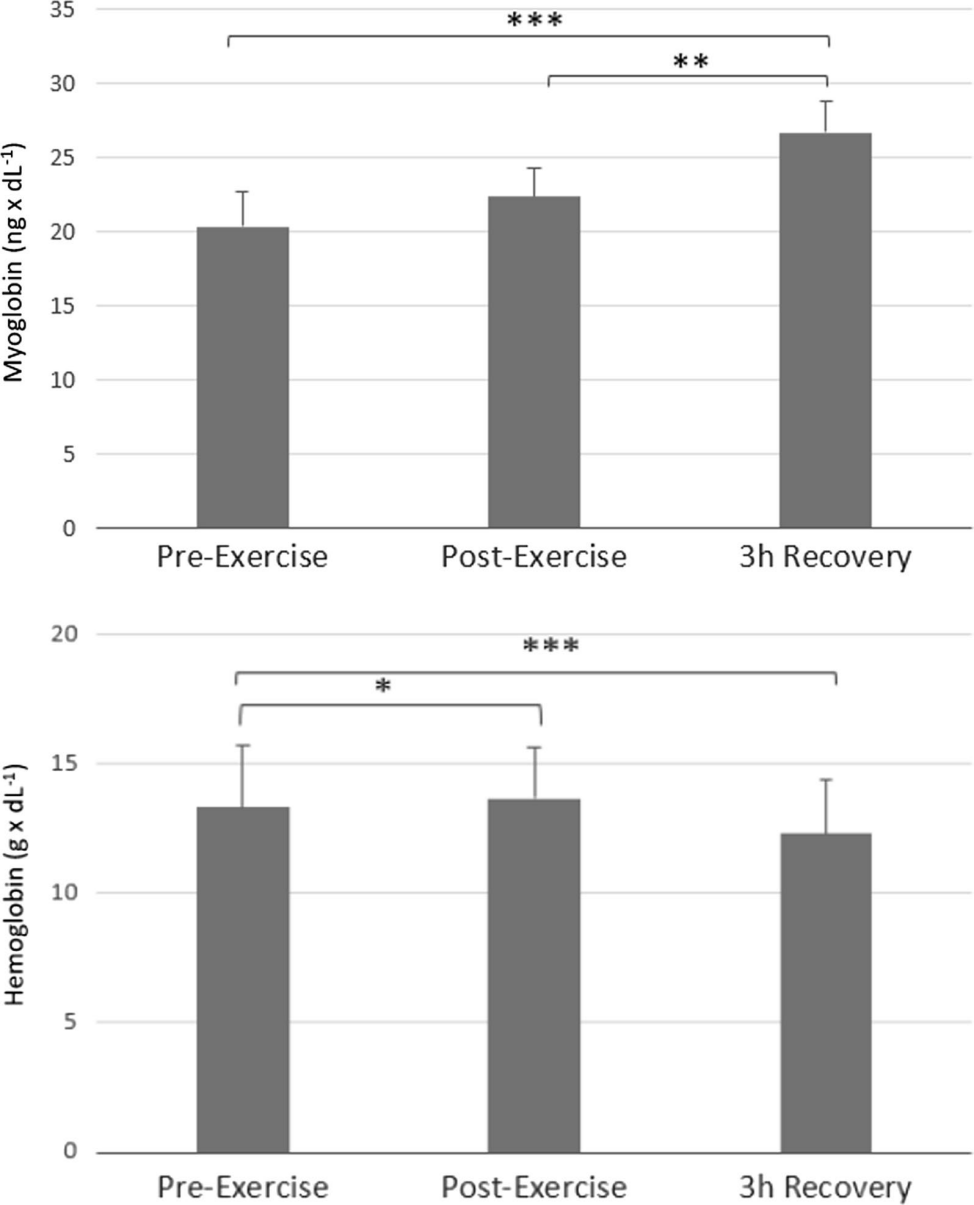
The effect of intense exercise on myoglobin and haemoglobin. *Note.* Values are presented as mean ± SD; Significant differences *p < .05; ***p < .001

A gradual and significant decrease in Hb concentration was observed (*p* < 0.05; Cohen`s d = 0.37 Pre-exercise vs. Post-exercise, *p* < 0.001, Cohen`s d = 1.03 Pre-exercise vs. 3 h Recovery), accompanied by a constant and significant increase of myoglobin (*p* < 0.001, Cohen`s d = 1.03, Pre-exercise vs. 3 h Recovery; *p* < 0.01, Cohen`s d = 0.68 Post-exercise vs. 3 h Recovery) (Fig. 4, Tables 3, 4).

**Table 3:** Changes in iron metabolism status during acute exercise

Parameter	Pre-exercise	Post-exercise	3 h Recovery	d Cohen		
				Pre-exercise vs Post-exercise	Pre-exercise vs 3 h Recovery	Post-exercise vs 3 h Recovery
Fe (µg x dL^−1^)	105.28 ± 38.38	135.06 ± 57.72^a^	101.18 ± 30.45^c^	0.62	0.12	0.77
Hepcidin (ng x dL^−1^)	16.01 ± 2.41	14.55 ± 1.95	15.16 ± 2.12	0.67	0.38	0.30
IL-6 (pg x mL^−1^)	1.77 ± 0.22	1.86 ± 0.17	1.68 ± 0.17	0.46	0.46	1.06
Transferin (mg x mL^−1^)	14.08 ± 0.33	14.14 ± 0.27	14.06 ± 0.32	0.20	0.06	0.27
TIBC (µg x dL^−1^)	335.86 ± 59.16	373.84 ± 90.88	322.89 ± 69.11^c^	0.51	0.20	0.64
Transferin saturation (%)	33 ± 14	40 ± 19^a^	32 ± 9	0.17	0.09	0.57
Myoglobin (ng x dL^−1^)	20.11 ± 5.26	22.07 ± 5.96^a^	26.62 ± 7.40^b,c^	0.35	1.03	0.68
Lactoferrin (ng x mL^−1^)	136.46 ± 20.26	86.54 ± 6.73	86.20 ± 5.51	3.70	3.90	0.06
Hemopexin (ng x mL^−1^)	902.88 ± 642.85	906.31 ± 706.31	918.07 ± 752.40	0.01	0.02	0.02
LDH (U x L)	266.47 ± 32.28	280.26 ± 46.01^a^	264.49 ± 29.75^c^	0.35	0.06	0.42
Ferritin (ng x mL^−1^)	90.78 ± 13.99	91.68 ± 15.20	89.54 ± 14.89	0.06	0.09	0.14
UIBC	237.45 ± 75.62	239.33 ± 98.18	218.13 ± 74.87	0.02	0.26	0.25

Values are presented as the mean ± SD

TIBC, total iron-binding capacity; LDH, lactate dehydrogenase; UIBC, unsaturated iron-binding capacity

^a^Pre-exercise vs. Post-exercise

^b^Pre-exercise vs. Recovery

^c^Post-exercise vs. Recovery (p < 0.05)

Effect size (Cohen`s d): 0.2 = small; 0.5 = medium; 0.8 = large

**Table 4:** Changes in RBC parameters during acute exercise

Parameter	Pre-exercise	Post-exercise	3 h Recovery	d Cohen		
				Pre-exercise vs Post-exercise	Pre-exercise vs 3 h Recovery	Post-exercise vs 3 h Recovery
HGB (g/dL)	13.34 ± 0.97	13.69 ± 0.93^a^	12.31 ± 1.03^b^	0.37	1.03	1.41
RBC (10^6^ × µL^−1^)	4.27 ± 0.32	4.40 ± 0.33^a^	3.97 ± 0.38^b,c^	0.40	0.86	1.21
HCT (%)	37.26 ± 2.51	38.21 ± 2.43^a^	34.17 ± 3.01^b,c^	0.38	1.12	1.49
MCV (fl)	87.49 ± 3.92	86.96 ± 3.95^a^	86.23 ± 3.82^b,c^	0.13	0.33	0.19
MCH (pg)	31.32 ± 1.55	31.16 ± 1.72	31.09 ± 1.78	0.10	0.14	0.04
MCHC (g x dL^−1^)	35.80 ± 0.48	35.82 ± 0.66	36.06 ± 1.14	0.04	0.32	0.27
RDW (%)	13.06 ± 0.72	13.36 ± 0.72	13.23 ± 0.74	0.42	0.23	0.18
MPV (fl)	8.048 ± 0.54	7.99 ± 0.55	7.79 ± 0.57^c^	0.11	0.47	0.36

Values are presented the mean ± SD

HGB, hemoglobin; RBC, red blood cell count; HCT, hematocrit; MCV, mean corpuscular volume; MCH, mean corpuscular hemoglobin; MCHC, mean corpuscular hemoglobin concentration; RDW, red cell distribution width; MPV, mean platelet volume;

^a^Pre-exercise vs. post-exercise

^b^Pre-exercise vs. recovery

^c^Post-exercise vs. Recovery (*p* < 0.05)

Effect – size (Cohen`s d): 0.2 = small; 0.5 = medium; 0.8 = large

For other parameters involved in iron metabolism, a significant decrease of lactoferrin was observed at the two time points after exercise compared to the baseline values ​​(*p* < 0.001, Cohen`s d = 3.7 Pre-exercise vs. Post-exercise, *p* < 0.001, Cohen`s d = 3.9, Pre-exercise vs. 3 h Recovery). The levels of LDH increased after exercise (*p* < 0.05, Cohen`s d = 0.35, Pre-exercise vs. Post-exercise), and then significantly decreased (*p* < 0.05, Cohen`s d = 0.42 Pre-exercise vs. 3 h Recovery) compared to the baseline values. No significant changes in ferritin concentration, transferrin, and UIBC saturation values were observed (Table 3).

Among the blood parameters examined, a significant increase in RBC after exercise was observed compared to the resting values (*p* < 0.05, Cohen`s d = 0.4 Pre-exercise vs. Post-exercise). During the restoration period, the RBC dropped significantly below the values ​​observed at rest (*p* < 0.001, Cohen`s d = 0.86, Pre-exercise vs. 3 h Recovery, *p* < 0.001, Cohen`s d = 1.49, Post-exercise vs. 3 h Recovery). Similarly, significant changes were observed for HCT (*p* < 0.05, Cohen`s d = 0.38, Pre-exercise vs. Post-exercise; *p* < 0.001, Cohen`s d = 1.12, Pre-exercise vs. 3 h Recovery; *p* < 0.001, Cohen`s d = 1.49, Post-exercise-Recovery); MCV (*p* < 0.05, Cohen`s d = 0.13, Pre-exercise vs. Post-exercise; *p* < 0.001, Cohen`s d = 0.33, Pre-exercise vs. 3 h Recovery; *p* < 0,001, Cohen`s d = 0.19, Post-exercise-Recovery); and MPV (*p* < 0.05, Cohen`s d = 0.36, Post-exercise vs. 3 h Recovery; *p* < 0.001, Cohen`s d = 0.47 Pre-exercise vs. 3 h Recovery) (Table 4).

**Fig. 5:**
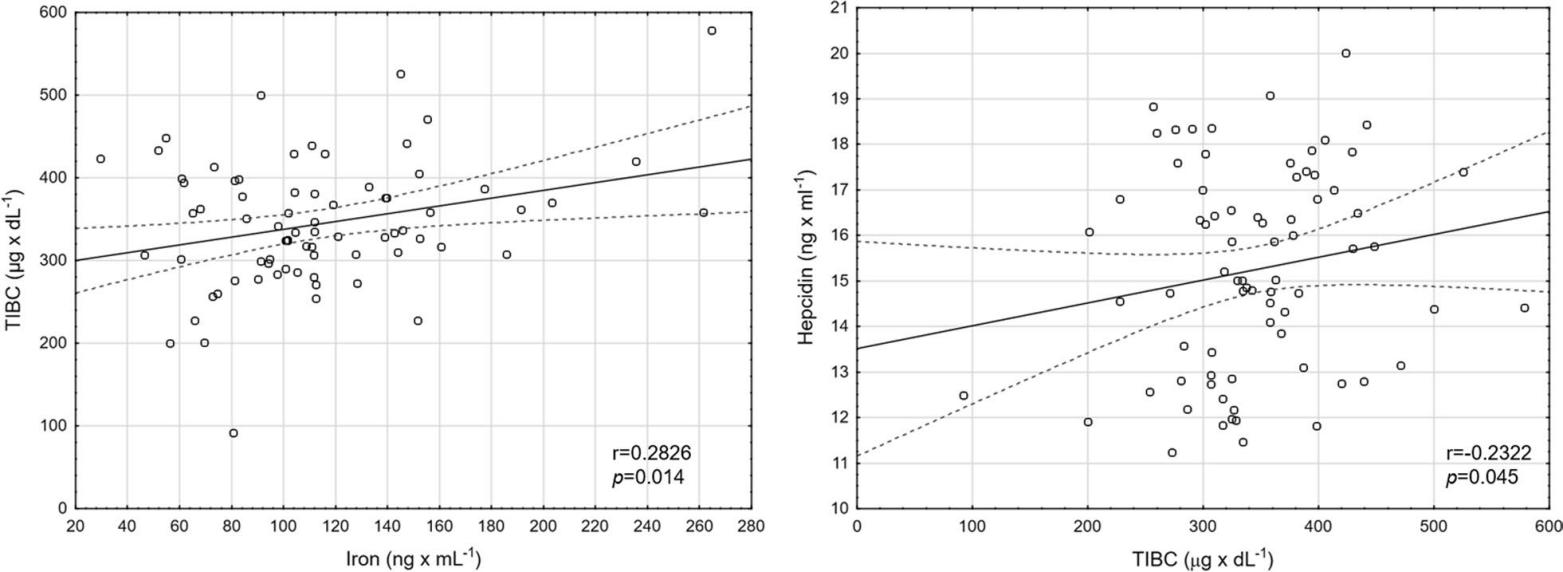
Correlation between iron and TIBC levels, and hepcidin and TIBC levels

Finally, TIBC was positively correlated with iron (r = 0.2826; *p* = 0.014), and negatively correlated with hepcidin (r = –0.2322; *p* = 0.045) (Fig. 5).

## Discussion

We here aimed to assess the impact of intense exercise performed at the end of the competition period when the low-load exercises are applied, and athletes have had the time to rest and recover after a heavy starting period on iron and iron regulatory proteins level. We found that intense exercise performed by rested athletes caused a short-term increase in iron ion levels, with a simultaneous increase in transferrin saturation and a slight increase in TIBC without a significant response from IL-6 or hepcidin.

The increased iron level after the exercise observed in this study indicates that the test used was a stimulus strong enough to elicit a hemolytic or non-hemolytic response. The increase of iron level after a bout of acute exercise was observed in a few studies, i.e., Schumacher et al. [27], Skarpańska-Stejnborn et al. [16], Dominguez et al. [23]. Moreover, the authors suggested that an increased iron levels as a result of vigorous exercise is one of the important factors responsible for triggering a sequence of reactions to regulate iron levels involving IL-6 and hepcidin.

In recent years, many studies have been conducted to determine the potential impact of intense exercise on hepcidin levels [4, 28]. The obtained data indicate that hepcidin levels are associated with an observable increase in iron levels and reach a maximum value 3–6 h after exercise. Hepcidin levels are also associated with a post-exercise increase in IL-6 levels [28]. According to Newlin et al. [29], IL-6 levels correspond to the release of hepcidin during exercise-induced inflammation. However, this phenomenon was not confirmed in the current study, as we observed a significant post-exercise increase in iron levels, with a simultaneous small, non-significant increase in IL-6 and lack of hepcidin responses. The lack of a significant increase in IL-6 levels after the exercise test was surprising but not implausible.

Literature data suggested that plasma Il-6 concentration may increase up to 100 fold after exercise. Moreover, the concentration of pro-inflammatory cytokines, including IL-6, in the serum significantly increased after a long-term endurance effort, e.g., a marathon or triathlon [30, 31]. This response may be significantly less pronounced after a short vigorous exercise [32]. Cullen et al. [33] have shown that IL-6 response on acute exercise induces comparatively small and immediate post-exercise IL-6 increase. Additionally, exercise training may reduce basal IL-6 production as well as the magnitude of the acute exercise IL-6 response. A decreased plasma IL-6 concentration at rest as well as in response to exercise appears to characterize normal training adaptation [34]. Considering existing literature data and results of our study it appears that IL-6 magnitude may depend on many factors such as the training level of athletes, intensity, and/or duration of exercise.

In addition, the training frequency of elite athletes is often so high that the short time between consecutive sessions may not be sufficient to achieve full recovery; this, in turn, may lead to the accumulation of harmful metabolites and an increase in the inflammatory response [35]. It is worth emphasizing that the current study involved young, but highly trained athletes. It has been proved that trained individuals have a lower level of IL-6 in serum [34]. Furthermore, our study was conducted at the final stage of the starting period, when the exercise load was reduced. Athletes who participated in the study had time to rest and recover after basketball gaming season.

The lack of significant changes in IL-6 levels may be responsible for the lack of hepcidin level increase observed in other studies. It is also important to note that trained athletes show a specific adaptation to exercise, i.e., increased resting levels of hepcidin. Sandström et al. [36] compared groups of training and non-training women and showed that the resting hepcidin levels in the training women were approximately 40% higher than those in non-training women, indicating that the higher values ​​in training individuals are a result of training adaptation. Similar to our findings, no significant changes in IL-6 and hepcidin levels were observed by Troadec et al. [10] after a 45-min submaximal ergometer test of healthy volunteers. In turn, Kasprowicz et al. [37] reported a significant increase in IL-6 levels in athletes after a 100 km run (ultra-marathon), which was not accompanied by an increase in hepcidin levels. A major involvement of hepcidin in the regulation of iron homeostasis was also demonstrated by Peeling et al. [38], who showed that changes in hepcidin levels post-exercise depend not only on the duration of exercise or IL-6 levels, but also on the initial serum iron and ferritin levels. In athletes with lower baseline iron and ferritin levels, the increase in hepcidin was significantly lower than that in athletes with higher baseline iron and ferritin levels. Whereas the results of the current study suggest that the observed significant increase in post-exercise iron levels is not a strong enough stimulus to activate hepcidin dependent mechanism of iron regulation in well-rested athletes.

The disturbance of iron metabolism, resulting from the presence of inflammation manifested by increased IL-6 levels, is, as mentioned above, associated with training periods characterized by high loads (preparation and competition period). In the current study, all measurements were conducted at the final stage of the starting period. This stage of training is characterized, as already mentioned, by a reduced training load and, therefore, may impact the biochemical and morphological parameters of the subjects.

Ostojic and Ahmetovic [39] showed that in highly trained athletes (footballers) during the preconditioning period, most of the parameters related to iron management are higher than those in the playing season associated with higher loads and, thus, fatigue. Importantly, Banfi et al. [11] observed seasonal variability of iron metabolism parameters related to different training loads. It can be assumed that the management of iron ions during the restitution period may be regulated by different mechanisms than those operational during intense training or competition period.

We did not note any changes in ferritin levels in the current study. Changes in ferritin levels, especially their reduction, are frequently observed among athletes and indicate iron store depletion [40]. Ferritin levels positively correlate with tissue iron stores, including the bone marrow, and hence, they are a good indicator of iron reserves in the body [41]. Since muscle oxygen consumption increases during intense exercise, which increases ROS levels, it is likely that ROS may contribute to the release of some of the iron stored in ferritin [42].

An interesting observation of the current study was the lack of changes in transferrin levels during the analyzed period, with a simultaneous change in the parameters directly related to its activity, i.e., TIBC and transferrin saturation after exercise. Transferrin saturation significantly increased immediately after exercise, while changes in TIBC (reflecting changes in iron levels) increased immediately after exercise and then significantly decreased after a 3 h rest period. In addition, we observed a significant positive correlation between TIBC and iron levels, and a negative correlation between TIBC and hepcidin. Typically, 60% of the serum transferrin pool is in the form of apotransferrin, i.e., a protein that is not saturated with iron. Apotransferrin is responsible for capturing free iron ions, including those from damaged hemoglobin [43]. The increase in transferrin saturation and changes in TIBC levels observed in the current study in parallel with the increased iron levels may indicate that iron capture by apotransferrin is the main mechanism responsible for the reduction of serum iron levels to the baseline values during the restitution period. The decrease in TIBC observed after a 3 h rest suggests that the iron levels have returned to the baseline values.

According to Hymes et al. (1986), a TIBC increase in athletes suggests an increased need for iron. The TIBC values increase with decreasing iron stores indicating that the iron is being moved to the stored iron pool.

Considering that the observed changes in iron ion level occurred within 3 h of exercise, it can be concluded that capturing iron by apotransferrin is a relatively rapid mechanism of iron level regulation and may be related to the high training level of the participants. The iron released during exercise was entirely captured by the circulating transferrin in the serum, without the need to activate additional regulatory mechanisms, e.g., increasing the hepcidin levels. Changes in transferrin saturation or TIBC are often used to characterize the dynamic changes in iron levels during training, or to observe the effects of a single, intense exercise [44, 45]. However, the reports on TIBC and transferrin saturation in athletes are inconsistent. Whereas we have observed changes in TIBC similar to those reported here in a previous study involving a group of rowers [16], others have observed the involvement of the IL-6–hepcidin axis. Deruisseu et al. (2004) reported no changes in iron levels or parameters characterizing its economy, such as transferrin, TIBC, or transferrin saturation, in participants after 3 weeks of strength training. On the other hand, Podgórski et al. [46] demonstrated a simultaneous increase in TIBC and iron levels throughout the annual training cycle of field hockey players.

Since iron deficiency, often observed in athletes, may negatively impact not only the oxygen transport and immune defenses, but also the individual’s sports performance, monitoring these parameters during training is important [9].

Lactoferrin is another protein that regulates the concentration of iron ions in the body. Its levels rapidly increase in response to inflammation [47]. The data presented herein indicated lack of inflammation in the study participants in the first few hours after exercise, which would explain the lack of the expected increase in lactoferrin levels after exercise.

The iron balance is also characterized by red cell parameters, such as RBC, HTC, Hb, and myoglobin. In a study of Boyadjiev and Tarałow [48], the values ​​of hematological variables in trained women were lower than those in the untrained control group. In addition, they observed differences in these values ​​among athletes from different disciplines, with the lowest values noted for representatives of high-intensity training disciplines, including rowers and swimmers. The intense physical effort that the basketball players were subjected to in the current study resulted in a significant but short-term increase in RBC, Hb, and HTC, and their levels were comparable with the baseline values during the 3 h rest period. The hemolysis that occurs during intense exercise may lead to disturbances in both, hematological parameters, and iron metabolism, leading to iron deficiency. Hence, iron supplementation is often recommended for athletes, especially for women, even in the absence of anemia symptoms [49, 50].

The observed increase in RBC and Hb values after physical exertion, along with simultaneously increased iron levels, may indicate a response that is not disturbed by inflammation. They may also suggest that the initial iron levels in the group of female players selected for the current study were sufficient and stable.

## Conclusions

Intense exercise performed by highly qualified athletes during the training period focused on regeneration caused a short-term increase in iron ion levels, with a simultaneous increase in transferrin saturation and a slight increase in TIBC. Both parameters returned to baseline values during the 3 h recovery period. However, no changes in the levels of IL-6 and hepcidin, and transferrin and ferritin, were observed. Post-exercise increase in hematological parameters suggests that ferritin, and not post-exercise hemolysis, may be the source of iron ions. This could indicate that the study participants were not iron-deficient.

Considering the study results within the context of existing literature it seems that the threshold level required for the induction of Il-6 and hepcidin response in highly trained, but rested athletes is higher than in athletes during their routine training program.

### Study limitations

Lack of elaboration of the potential relationships between menstrual cycle and study results.

## Data Availability

Due to ethical concerns the datasets generated and/or analyzed during the current study supporting data cannot be made openly available however, are available from the corresponding author on reasonable request.

## Abbreviations

RBC: Red blood cell count
Hb: Hemoglobin
HR_max_: Maximal heart rate in beats per minute
HTC: Hematocrit
MCH: Mean corpuscular hemoglobin
MCHC: Mean corpuscular hemoglobin concentration
MCV: Mean platelet volume
MPV: Mean platelet volume
PLT: Platelet count
RDW: Red cell distribution width
RER: Respiratory exchange ratio
TIBC: Total Iron Binding Capacity
UIBC: Unsaturated iron-binding capacity
bpm: Heart rate (beats per minute)
LDH: Lactate dehydrogenase
Vmax: Maximal run velocity in kilometers per hour
VO_2_max: Maximal oxygen consumption
